# Lenalidomide and dexamethasone in patients with relapsed multiple myeloma and impaired renal function: PrE1003, a PrECOG study

**DOI:** 10.1038/s41408-018-0110-7

**Published:** 2018-08-29

**Authors:** Joseph Mikhael, Judith Manola, Amylou C. Dueck, Suzanne Hayman, Kurt Oettel, Abraham S. Kanate, Sagar Lonial, S. Vincent Rajkumar

**Affiliations:** 1 Mayo Clinic, Phoenix, AZ USA; 2 International Myeloma Foundation, Los Angeles, CA USA; 3 Dana-Farber Cancer Institute, Boston, MA USA; 4 Mayo Clinic, Rochester, MN USA; 5 Gundersen Health System, La Crosse, WI USA; 6 West Virginia University Mary Babb Randolph Cancer Center, Morgantown, WV USA; 7 Emory University Winship Cancer Institute, Atlanta, GA USA

## Abstract

Renal insufficiency is common in patients with relapsed multiple myeloma and can often limit choice of therapy. Lenalidomide, a critical agent in the treatment of relapsed multiple myeloma, is renally cleared., This phase I/II trial evaluated the efficacy and safety of lenalidomide with dexamethasone in patients with relapsed multiple myeloma and renal insufficiency. Three groups were treated, with creatinine clearance 30–60 cc/hr (group A), CrCl < 30 not on dialysis (group B), and patients on dialysis (group C) at escalating doses of lenalidomide. A total of 63 patients were treated and no DLTs were observed in phase I. All three groups were able to escalate to full dose lenalidomide 25 mg daily 21/28 days, although due to reduced accrual the phase II component was not entirely completed for groups B and C. Adverse events were as expected, including anemia, diarrhea and fatigue. Ten patients experienced grade 3–4 pneumonia. Overall response rate was 54% across all groups. PFS was 7.5 months and OS was 19.7 months. Lenalidomide can be given at full dose 25 mg daily 21/28 in patients with a CrCl > 30, and can be given daily to those with CrCl < 30, even when on dialysis, at doses of at least 15 mg daily.

## Introduction

Multiple myeloma is diagnosed in approximately 30,000 Americans annually^1^ and remains an incurable hematologic malignancy characterized by frequent early response followed by universal treatment relapse necessitating multiple sequential therapeutic regimens^2^. The most commonly used backbone regimen for relapsed myeloma employs lenalidomide and dexamethasone, eiher alone or in combination with other novel agents or conventional chemotherapy^3^.

Myeloma is more common among African-Americans than among white patients (incidence rate of 13 cases/100,000 vs. 5.6 cases/100,000). African-Americans are, in turn, at higher risk for chronic kidney disease^4^. Lenalidomide, while highly effective, is known to be substantially excreted by the kidneys, and the risk of toxic reactions to this drug may be greater in patients with impaired renal function^5^. Dysuria, renal failure, hematuria, acute renal failure, azotemia, calculus ureteric, and renal mass are listed as reported adverse events in the package insert.

Chen et al.^6^ conducted a multi-center study with lenalidomide 25 mg a day as a single oral dose in five groups of subjects defined by renal function: (1) normal (CrCl > 80 mL/min); (2) mild (CrCl: >50–<80 mL/min); (3) moderate (CrCl: >30–<50 mL/min); (4) severe (CrCl < 30 mL/min, but not on dialysis); (5) end stage renal disease (ESRD)(requiring dialysis). Thirty subjects over the age of 35 were included in the study. Subjects with normal, mild, moderate, or severe renal insufficiency received a single 25 mg oral dose of lenalidomide. Subjects with ESRD received two single 25 mg doses, separated by 7–10 days: one dose on a non-dialysis day and the other dose 3 h before a 4-h hemodialysis. This study demonstrated that patients with renal insufficiency could be treated safely and effectively with reduced dosing. Based on modeling to produce AUCs in patients with renal insufficiency that were comparable to those produced by full dose in patients with normal renal function, the following doses were recommended:

Renal function (CrCL) multiple myeloma

- Mild (CrCl ≥ 50 mL/min) 25 mg qd (Full Dose)
- Moderate (30 ≤ CrCl < 50 mL/min) 10 mg qd*
- Severe (CrCl < 30 mL/min, not requiring dialysis) 15 mg q 48 h
- ESRD (CrCl < 30 mL/min, requiring dialysis) 15 mg 3 × a week following each dialysis

*The dose may be escalated to 15 mg qd after two cycles if patient is tolerating drug well but not responding to treatment

Since data on the maximum tolerated dose of lenalidomide in patients with impaired renal function is lacking and this remains a clinically significant issue, we undertook this phase I/II trial in previously treated patients with multiple myeloma and varying degrees of renal impairment. The goal of the phase I component was to clinically assess the MTD (maximum tolerated dose) per risk group. In the phase II component, efficacy was assessed. This trial was designed after the Chen et al.^6^ experience.

## Subjects and methods

### Patient selection

Subjects with previously treated multiple myeloma were eligible to participate. They must have had measurable disease, determined by one of the following assessed within 21 days prior to registration: serum monoclonal (M) protein ≥ 1 g by protein electrophoresis; urine m protein ≥ 200 mg on 24 h electrophoresis; serum immunoglobulin free light chain ≥10 mg/dL with abnormal serum immunoglobulin kappa to lambda free light chain ratio; or monoclonal bone marrow plasmacytosis ≥30% (considered evaluable disease). If both serum and urine m-components were present, then both were required to be followed for response evaluation. Patients must have had ECOG performance status of 0, 1, or 2, and must have completed previous therapy at least 2 weeks prior to study entry. Patients were required to have renal impairment at baseline, defined as serum creatinine clearance ≤60 mL/min, measured within 21 days prior to registration. Other organ and marrow function was required to be acceptable (absolute neutrophil count ≥ 1000 cells/mm^3^, platelet count ≥ 75,000 cells/mm^3^, total bilirubin ≤ 2 mg/dL, AST (SGOT) and ALT (SGPT) ≤ 3 times the institutional upper limit of normal.) Pregnant women were not eligible, nor were females of childbearing potential unwilling to use two forms or contraception or men unwilling to wear a latex condom during sexual contact. Patients who had previously received lenalidomide were eligible if they had experienced a clinical response of any duration or a progression-free interval of at least 6 months from the start of that therapy. HIV-positive patients on combination antiretroviral therapy were not eligible, nor were patients with known hypersensitivity to thalidomide or other immunomodulatory drugs, patients with a history of Stevens-Johnson syndrome, patients requiring concurrent radiation therapy (other than for palliation of a single bone lesion or fracture), or patients with another active malignancy.

### Registration

All patients provided signed, written informed consent. During both phase I and phase II, patients were enrolled concurrently into groups based on the degree of renal impairment. Registration was completed through a web-based data capture system. During phase I, registration was preceded by telephone contact to assure that a place on the protocol was available.

### Treatment administration

Treatment began within seven working days after registration and consisted of lenalidomide at the assigned dose as shown in Table 1, dexamethasone 40 mg orally on days 1, 8, 15, and 22 of a 28 day cycle, and anticoagulation. Anticoagulation consisted of aspirin at either 81 mg/day or 325 mg/day at the physician’s discretion. Heparin, low molecular weight heparin, or Coumadin could be used if the patient was intolerant to aspirin.

**Table 1:** Renal function groups, dose levels, and cycles administered

Renal function groups and dose levels						
	Group A		Group B		Group C	
	CrCl 30–60 mL/min		CrCl < 30 mL/min, not on dialysis		CrCl < 30 mL/min, on dialysis	
Dose level	Dose (mg)	Days	Dose (mg)	Days	Dose (mg)	Days
1	10	1–21	15	Every other day, days 1–21	15	3 × /wk, days 1–21
2	15	1–21	25	Every other day, days 1–21	10	1–21
3	25	1–21	15	1–21	15	1–21
4	–	–	25	1–21	25	1–21
Number of cycles administered						
Dose level	*n*	Median (range)	*n*	Median (range)	*n*	Median (range)
1	6	16.5 (2–48)	3	5 (2–10)	3	3 (3–4)
2	3	4 (2–23)	3	3 (3–24)	3	7 (3–34)
3	6	11 (3–47)	3	5 (3–35)	3	5 (1–27)
4	–		8	3.5 (1–24)	5	3 (2–6)
Expansion group	14	6 (1–20)	2	7.5 (6–9)	–	
Total patients	29		19		14	

### Dose escalation phase: definition of dose limiting toxicity

Dose limiting toxicity (DLT) was defined as any of the following events determined by the investigator to be possibly, probably, or definitely related to lenalidomide within the first cycle of therapy irrespective of whether the adverse events resolved:

- Grade 3 or higher neutropenia with fever ≥ 38.5 °C
- Grade 4 neutropenia ≥ 7 days
- Grade 4 or higher thrombocytopenia
- Other non-hematologic grade 4 or higher adverse event not present prior to starting therapy or not due to underlying cause

In order to be evaluable for consideration of DLT, a subject must have received at least one dose of both lenalidomide and dexamethasone. Subjects who withdrew before the end of cycle 1 for reasons other than adverse events were to be replaced.

A standard 3 + 3 design was employed. First, three patients were accrued to each group at a dose level, starting with dose level 1. If zero of three patients experienced a DLT, then enrollment continued at the next higher dose level. If two patients experienced a DLT, then the recommended dose level would have been exceeded. If one patient experienced a DLT, then three more patients were accrued to the group and dose level. If no additional patients experience DLT, then enrollment continued at the next higher dose level. If any patients among the additional three (total of two out of six) experienced DLT, then the recommended dose would have been exceeded. The recommended phase II dose was the highest dose at which fewer than two of six patients experienced DLT.

Post-cycle 1 lenalidomide and dexamethasone dose modifications were specified in the protocol, as were minimum ANC and platelet levels prior to treatment on day 1 of each cycle. If patients in groups B or C had improvement in renal function, they could receive a one-time escalation in lenalidomide dose level after two cycles of treatment had been completed. The protocol specified criteria for ancillary treatment with growth factors, infection prophylaxis, GI prophylaxis, and anti-coagulation therapy.

Adverse events were carefully and routinely monitored using the National Cancer Institute Common Terminology Criteria for Adverse Events (CTCAE), Version 4.0. Events of grade 1 and 2 that were deemed possibly, probably or definitely related to treatment and all grade 3–4 adverse events were to be reported via case report forms. All deaths within 30 days of the patient’s last study treatment were to be reported, regardless of attribution, as well as deaths after 30 days that were at least possibly related to treatment. Serious adverse events were required to be reported within 24 h of discovery. Pregnancy of a female subject or the female partner of a male subject was considered a serious adverse event.

Patients remained on treatment until disease progression, withdrawal of consent, or adverse events that in the opinion of the investigator precluded further participation.

### Study monitoring

The study was conducted under the oversight of each participating institution’s local Review Board. During the phase I component of the study, weekly teleconference calls were held to share information about accumulating adverse events and general study conduct. The phase II component of the study was monitored and reviewed semi-annually by the PrECOG Data Safety Monitoring Board. The trial is registered in clinicaltrials.gov (NCT00790842).

### Efficacy evaluation

Myeloma response criteria defined by the International Myeloma Working Group were used to determine response.

## Statistical considerations

The study was designed to have three independent single-arm phase I trials to determine the recommended phase 2 dose, followed by enrollment of 15 patients to each trial for assessment of efficacy. The phase 1 groups each followed a standard 3 + 3 design as described above. This design provided a low probability of escalating if the true DLT rate was high (17% for true DLT rates of 50%) and a high probability of escalating if the true DLT rate was low (91% for a DLT rate of 10%).

For the phase 2 component, the primary endpoint was the proportion of patients who have at least a partial response to treatment. The analysis was to include the six patients treated at the recommended phase 2 dose in each dose group, along with the additional patients enrolled to the efficacy group for that dose group. The denominator in estimating rates included all eligible, treated patients. All dose groups were to be combined for the efficacy analysis, so a total of 63 eligible, treated patients were planned for analysis. The study was designed to distinguish a favorable response rate of 55% from a rate of 40% not considered favorable. The decision rule stated that the regimen would be considered efficacious if 31 or more patients had a partial response or better. This design provided 85% power to distinguish a favorable response rate of 55% from a rate of 40%, using a one-sided test with 9% type 1 error.

Due to slower than anticipated accrual to the efficacy group, a revised exploratory statistical analysis plan was derived prior to final analysis. This plan would declare the treatment worthy of further study if 18 or more of the 34 patients responded to treatment. This design had 84% power to distinguish a response rate of 60% from a null rate of 40%, using a one-sided test with 10% type I error.

Overall survival was defined as the time from registration to death or last contact. Progression-free survival was defined as time from registration to documented progression or to treatment discontinuation due to progression. Patients without progression were censored at the date of last disease assessment. Patients without disease assessment follow-up dates post treatment were censored at the last treatment date.

Response was classified into nine categories, ranging from stringent complete response to progression. In order to be considered a response for the efficacy analysis, a classification of partial response or better was required. Patients without follow-up disease assessments were considered unevaluable, and were included in the denominator when calculating the response rates.

Descriptive statistics were used to characterize patients at study entry. Exact binomial confidence intervals (90%) were computed for the response rates. The method of Kaplan and Meier was used to estimate progression-free and overall survival. Medians and 90% confidence intervals were estimated for PFS (progression free survival) and OS (overall survival). Mehta’s exact test for ordered categorical data (1984) was used to compare highest degree toxicity rates among groups. Stata (version 14.2) was used for all analyses.

## Results

**Fig. 1:**
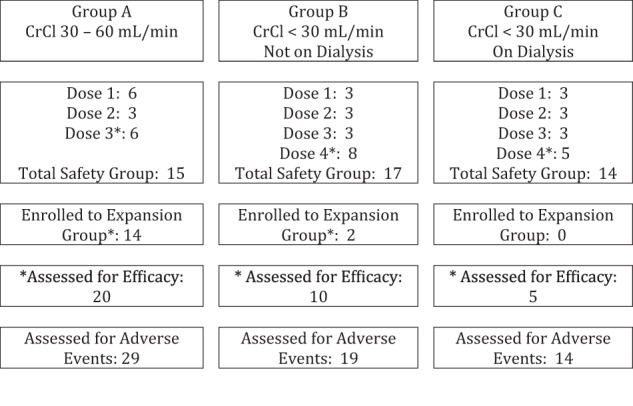
Disposition of cases

A total of 63 patients were enrolled between January 2009 and November 2015 from 12 different institutions (nine institutions during phase 1). During the phase 1 component, one patient was found to be ineligible after enrollment but prior to treatment and was excluded from the analysis. Thus, the analysis group includes 62 patients. Table 1 and Fig. 1 show the distribution of patients among renal function groups and dose levels, along with analysis cohorts for safety and efficacy.

Table 2 shows patient characteristics at baseline, by renal function group and overall. Half of the patients were female and 84% were Caucasian. Median age was 71.5 (range, 48–89). Over half of the patients had received at least 2 prior treatment regimens.

**Table 2:** Patient Characteristics

		Group A (*n* = 29)		Group B (*n* = 19)		Group C (*n* = 14)		Total (*n* = 62)	
		*n*	%	*n*	%	*n*	%	*n*	%
Creatinine clearance	Median	42.6		22.8		11.5		27.4	
	Range	30.0–68.4		2.3–29.7		4.1–32.4		2.3–68.4	
	IQR	37.0–51.2		15.7–25.4		9.7–17.2		15.7–42.1	
	Missing	2		1				3	
Serum M-spike (g/dL)	<1	5	17.2	12	63.2	7	50.0	24	38.7
	>=1	24	82.8	7	36.8	7	50.0	38	61.3
Urine M-spike (mg/24 h)	<200	15	51.7	8	42.1	11	84.6	34	55.7
	>=200	14	48.3	11	57.9	2	15.4	27	44.3
Serum IgFLC (mg/dL)	<10	5	17.2	2	10.5	0	0	7	11.3
	>=10	24	82.8	17	89.5	14	100.0	55	88.7
Bone marrow plasma cells (%)	<30	16	55.2	11	57.9	7	50.0	34	54.8
	>=30	13	44.8	8	42.1	7	50.0	28	45.2
Beta-2 microglobulin (mg/L)	<3.5	3	10.3	1	5.3	1	7.1	5	8.1
	>=3.5–<5.5	7	24.1	5	21.1	1	7.1	13	19.4
	>=5.5	19	65.5	13	68.4	11	78.6	43	69.4
Albumin (g/dL)	<3.5	11	37.9	7	36.8	8	57.1	26	41.9
	>=3.5	18	62.1	12	63.2	6	42.9	36	58.1
Type of dialysis	None	28	96.6	17	89.5	0	0	45	72.6
	Peritoneal	0	0	0	0	2	14.3	2	3.2
	Hemo-dialysis	1	3.5	2	10.5	12	85.7	15	24.2
Number of prior therapies	1	15	51.7	10	52.6	5	35.7	30	48.4
	2–3	8	27.6	8	42.1	6	42.9	22	35.5
	4 or more	6	20.7	1	5.3	3	21.4	10	16.1

*IQR* Interquartile Range

Table 1 provides a summary of the number of cycles administered, by dose level and renal function group. Seventeen patients remained on treatment for more than 12 cycles.

During the phase I component of the study, no DLTs were observed. Thus, the highest dose level tested in each renal function group was selected for further evaluation. Table 3 shows highest degree adverse events experienced by each patient, tabulated by renal function group and dose level. Twenty-eight patients experienced grade 3 or 4 treatment-related events. One patient in group C (the dialysis group) assigned to dose level 2 experienced a lethal adverse event reported as lung infection, sepsis, and multi-organ failure. Five unrelated lethal events were reported: cirrhosis, intra-abdominal hemorrhage, sudden death, and ESRD (*n* = 2).

**Table 3:** Highest degree treatment-related adverse events per patient, by renal function group and dose level

			Highest degree treatment-related					
Group	Dose level	Pts	None	1	2	3	4	5
A	1	6	3		2	1		
	2	3	1			1	1	
	3	6	1	1		4		
	Expansion	14	1	4	3	6		
B	1	3	1			1	1	
	2	3	1			1	1	
	3	3		1	1	1		
	4	8	1	1	2	3	1	
	Expansion	2		1			1	
C	1	3	3					
	2	3		1		1		1
	3	3		2		1		
	4	5	2			3		
TOTAL		62	14	11	8	23	5	1

Most common treatment related adverse events were anemia, decreased appetite, and muscle weakness/fatigue. Additionally, 11 episodes of grade 3 and 1 grade 4 pneumonia were reported for ten patients across all groups and dose levels; four were considered possibly related to treatment. Other grade 4 events, each occurring in a single patient, were atrioventricular block, decreased blood calcium, myocardial infarction, pancytopenia, peripheral arterial occlusive disease, and septic shock.

As previously described, six deaths on study were noted. One death from unknown cause within 30 days of the end of treatment was also reported.

Supplementary Table 1 shows these same adverse events broken down by two factors of interest: whether or not the patient was assigned to a dose of at least 15 mg per day, and whether or not the patient was assigned to a daily dosing group. The distribution of highest degree adverse events did not differ when comparing patients who were assigned doses of at least 15 mg/day and those assigned smaller doses (Fisher’s exact *p* = 0.28).

The distribution of highest degree adverse events did not differ when comparing patients who were assigned to daily doses and those assigned less frequent doses (Fisher’s exact *p* = 0.44).

Table 4 shows best overall response among 35 patients who received the recommended phase II dose for their renal function group. Across all groups, there were 19 responses, giving a rate of 54.3% (90% exact bionomial confidence interval, 39.2–68.8%). This met the criteria for success specified a priori using the revised analysis plan. Response by dose and frequency can be viewed in Supplementary Table 2.

**Table 4:** Best overall response—efficacy group

	Group A	Group B	Group C	Total
Efficacy group (patients treated at phase 2 dose)	20	10	5	35
Response rate *n* (%)	12 (60.0%)	6 (60.0%)	1 (20.0%)	19 (54.3%)
90% CI	39.4–78.3%	30.4–85.0%	1.0–65.7%	39.2–68.8%

There was no difference in response rate associated with dose (<15 vs. ≥15 mg/day, one-sided exact *p* = 0.43) or frequency (daily vs. less frequently, one-sided exact *p* = 0.62).

**Fig. 2:**
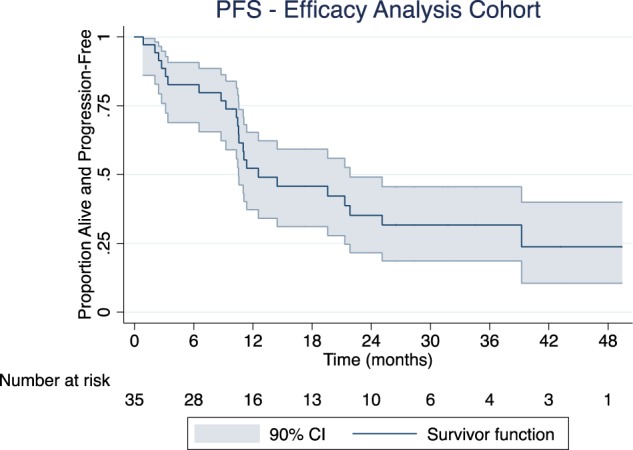
Progression-free survival

Figure 2 shows progression-free survival among patients in the efficacy analysis group. Median PFS was 12.6 months (90% confidence interval, 10.5 to 21.8 months). There were 43 PFS events overall, 23 of which were among patients in the efficacy analysis group. Twelve of these were progression events, five in the efficacy analysis group.

**Fig. 3:**
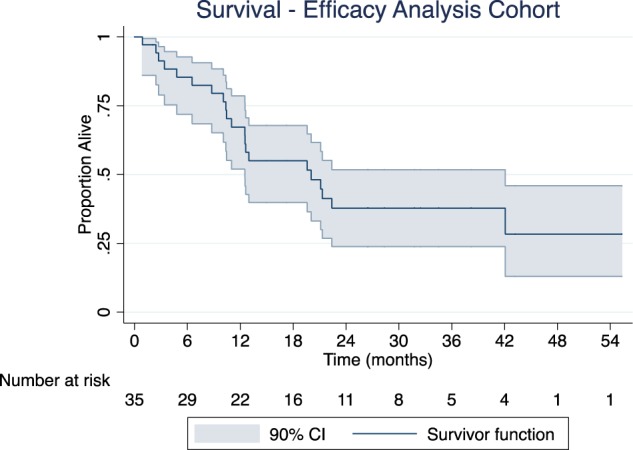
Overall survival

Figure 3 shows overall survival among patients in the efficacy analysis group. At the time of study closure, 37 deaths had occurred, 21 in the efficacy analysis group. Median overall survival was 20.0 months (90% confidence interval, 12.5–42.1 months).

## Discussion

Renal dysfunction is common, ranging from 20–50% in myeloma and becomes increasingly important and more aggressive in relapsed disease^7–9^. Indeed, one of the many factors that heavily influences therapy selection in relapsed multiple myeloma is renal function, due to its prognostic influence, its comorbidity to the patient and in the metabolism of anti-myeloma agents. It is also important to note that many other non myeloma factors can contribute to renal insufficiency in patients with myeloma; in a series of nearly 200 patients who underwent renal biopsy with myeloma and renal failure, 15% had causes of renal failure that were not myeloma related, including arterionephrosclerosis, diabetic nephrosclerosis, postinfectious glomerulonephritis, and smoking related gloomerulopathy^10^. Other plasma cell disorders associated with renal compromise must also be excluded, including amyloidosis and immunoglobulin deposition disease.

Although many combinations are used in relapsed myeloma, one of the key agents employed is lenalidomide. It can be paired with dexamethasone alone^11,12^, but more recently has been used in triplet therapy with dexamethasone plus carfilzomib^13^, ixazomib^14^ daratumumab^15^, and elotuzumab^16^. It has also been combined with more conventional chemotherapy such as cyclophosphamide^17^ and bendamustine^18^.

Due to its renal clearance, clinicians are not well versed in dosing of lenalidomide in patients with renal insufficiency, and may indeed be under-dosing or even overdosing patients—hence the need of the current study to more clearly delineate the appropriate dosing of lenalidomide.

As expected, the response rate was approximately 50% in this high risk group of patients with renal insufficiency in a real world setting. The response rate in the original phase 3 trial of lenalidomide and dexamethasone was 50–63% in all renal subgroups, although the rate of thrombocytopenia and dose delays/interruptions were higher in the severe renal insufficiency group^19^. This has been replicated in smaller studies of patients, where lenalidomide and dexamethasone were given to patients with renal insufficiency, including patients on dialysis^20–23^ in each of these series, a small but significant number of patients experienced renal recovery.

It is somewhat surprising that in contrast to the product insert and earlier findings in small number of patients, we demonstrated that higher doses of lenalidomide could be used safely in patients with renal impairment. Indeed, those with a creatinine clearance of 30cc or greater (group A patients) could receive full dose therapy of 25 mg daily 21/28 days, just like patients with normal renal function. This is of great clinical utility as most patients do fall into this category of mild renal impairment and dose modifications are not necessary, simplifying the dosing schedule for clinicians.

This study also demonstrates the ability to continuously dose patients with lenalidomide with severe renal insufficiency (CrCl < 30 but not on dialysis—group B), with at least 15 mg and possibly 25 mg although too few patients were treated to be conclusive. Similarly, 15–25 mg daily can be given to patients on dialysis (group C), avoiding the inconvenience of three times weekly dosing. Overall, this simplifies the dosing to daily dosing in all patients independent of renal function with the caveat that dose reduction to 15 mg may be required in patients with severe renal insufficiency. We conclude this as over 20 patients were treated in both groups B and C daily and with doses of 15 mg or greater. This is consistent with the International Myeloma Working Group (IMWG) recommendations, which also add that monitoring for toxicity, namely myelosuppression should be considered more carefully in patients with renal insufficiency^24^.

The greatest limitation of this study was challenging accrual; with triplet combinations becoming more widely used in relapsed multiple myeloma it was more difficult to accrue to a doublet lenalidomide dexamethasone regimen. Nonetheless, the number of patients accrued is the largest in this group of patients and supports the conclusions drawn.

Future studies should be conducted to explore the ideal dosing of patients in triplet or even quadruplet combinations—although in most of those combinations there is a dose reduction in lenalidomide already.

## Key points

1. In patients with CrCl ≥ 30 full dose lenalidomide 25 mg daily 21/28 is feasible and effective
2. In patients with CrCl < 30 (on dialysis or not) lenalidomide can be given on a daily dosing regimen (not three times weekly)
3. In patients with CrCl < 30 (on dialysis or not) can be given lenalidomide at a dose of at least 15 mg daily 21/28

## Electronic supplementary material

Supplementary Tables
