# Supplementary material for: Lenalidomide and dexamethasone in patients with relapsed multiple myeloma and impaired renal function: PrE1003, a PrECOG study
Source: Blood Cancer J. 2018 Aug 29;8(9):86. doi: 10.1038/s41408-018-0110-7 (PMC6127323; doi:10.1038/s41408-018-0110-7)
Supplement: Supplementary file 1 — Supplementary Tables [file 41408_2018_110_MOESM1_ESM.docx]

Supplementary Tables

Table 1. Treatment-Related Adverse Events per Patient, by Dose and Frequency

|  |  |  | Highest Degree  Treatment-Related | | | | | |
| --- | --- | --- | --- | --- | --- | --- | --- | --- |
| Group | Dose Level | Pts | None | 1 | 2 | 3 | 4 | 5 |
| Dose | < 15 mg/day | 18 | 8 | 1 | 2 | 4 | 2 | 1 |
|  | > 15 mg/day | 44 | 6 | 10 | 6 | 19 | 3 |  |
| Frequency | Less than Daily | 9 | 5 |  | 2 | 2 |  |  |
|  | Daily | 53 | 9 | 11 | 8 | 21 | 3 | 1 |

Table 2. Best Overall Response – All Patients by Dose and Frequency

|  | Dose < 15 mg/day | Dose > 15 mg/day | Less than Daily Dosing | Daily  Dosing |
| --- | --- | --- | --- | --- |
| Patients | 18 | 44 | 9 | 53 |
| Response Rate n (%) | 11 (61.1%) | 24 (54.5%) | 5 (55.6%) | 30 (56.6%) |
| 90% CI | 35.7 – 82.8% | 38.8 – 69.6% | 21.2 – 86.3% | 42.3 – 70.2% |
